# Impact of intramyocardial hemorrage on LV remodeling after ST-elevation myocardial infarction

**DOI:** 10.1186/1532-429X-16-S1-P103

**Published:** 2014-01-16

**Authors:** Marc Sirol, Heger Gzara, Damien Logeart, Philippe Soyer, Eric Vicaut, Barnabas Gelen, Jean Jacques Mercadier

**Affiliations:** 1 Paris Sorbonne- Paris Cité; AP-HP Hôpital Lariboisière, Paris, France; 2 Unité de Recherche Clinique, AP-HP Hôpital Fernand Widal, Paris, France; 3 AP-HP Hôpital Bichat, Paris, France; 4 AP-HP Hôpital Henri Mondor, Paris, France

## Background

Left ventricle (LV) remodeling associated with lower LV ejection fraction after successfully treated ST-elevation myocardial infarction (STEMI) may occur in some patients. We investigated the prognostic value of intramyocardial hemorrhage (IMH) as assessed by T2* images beyond a comprehensive CMR assessment with late gadolinium enhancement (LGE) imaging including microvascular obstruction (MVO) evaluation.

## Methods

A total of 110 patients with STEMI were prospectively recruited and were examined with CMR at Day 4 ± 2 and Month 6 after reperfusion for measurement of LV end-diastolic (EDV) and end-systolic (ESV) volumes, LV ejection fraction (LVEF) infarct size (IS) and presence and extent of MVO and IMH. Adverse remodeling was defined as dilated left ventricular end-systolic volume indexes (EDV) at 6 months CMR.

## Results

All patients were analysed. 41 patients (45%) presented with Anterior AMI, 26 with Lateral (23%) and 29 with Inferior MI (32%). Mean age was 54 +/- 12 y.o (75% male). Mean delay for reperfusion therapy was 115 +/- 100 min. LV remodelling was observed in 39 patients (36%). Despite identical EDV, patients with LV remodelling had lower LVEF at baseline (45% +/- 7 vs 51 +/- 8, p < 0.01), a bigger IS (42 g +/- 20 vs 32 +/- 20; p < 0.01) and MVO extent (p < 0.01). By multivariate analysis, IMH (OR = 2.8[1.3-6.0]) and IS (OR = 3.2[1.8-12.5]) were identified as independent predictors of LV remodelling.

## Conclusions

Presence of IMH after STEMI assessed by T2* CMR predicts LV adverse remodeling.

## Funding

Research Grant from the french Society of cardiology (SFC)

